# Biomechanical Comparison of Shorts With Different Pads: An Insight into the Perineum Protection Issue

**DOI:** 10.1097/MD.0000000000001186

**Published:** 2015-07-24

**Authors:** Giuseppe Marcolin, Nicola Petrone, Carlo Reggiani, Fausto A. Panizzolo, Antonio Paoli

**Affiliations:** From the Department of Biomedical Sciences, University of Padova, Padova, Italy (GM, CR, AP); Department of Industrial Engineering, University of Padova, Padova, Italy (NP); and School of Sport Science, Exercise and Health, University of Western Australia, Crawley, Western Australia, Australia (FAP).

## Abstract

An intensive use of the bicycle may increase the risk of erectile dysfunction and the compression of the perineal area has been showed to be a major mechanism leading to sexual alterations compromising the quality of life. Manufacturers claim that pads contribute to increase cyclists perineal protection ensuring a high level of comfort.

To investigate the influence of various cycling pads with regard to perineal protection and level of comfort.

Nine club road cyclists rode 20 min on a drum simulator, located at the Nutrition and Exercise Physiology Laboratory, at a constant speed and gear ratio wearing the shorts with 3 cycling pads of different design and thickness: basic (BAS), intermediate (INT), and endurance (END). Kinematics and pressure data were recorded at min 5, 15, and 20 of the test using a motion capture system and a pressure sensor mat. The variables of interest were: 3-dimensional pelvis excursions, peak pressure, mean pressure, and vertical force. The comfort level was assessed with a ranking order based on the subjects’ perception after the 20-min trials and measuring the vertical ground reaction force under the anterior wheel as well as the length of the center of pressure (COP) trajectory on the saddle.

Results showed that the vertical force and the average value of mean pressure on the saddle significantly decreased during the 20-min period of testing for BAS and END. Mean peak pressure on the corresponding perineal cyclist area significantly increased only for BAS during the 20-min period. Interestingly objective comfort indexes measured did not match cyclists subjective comfort evaluation.

The lower capacity of BAS to reduce the peak pressure on the corresponding perineal area after 20 min of testing, together with its positive comfort evaluation, suggest that a balance between protection and perceived comfort should be taken into account in the choice of the pad. Hence, the quantitative approach of objective comfort indexes introduced in this study could be helpful for manufacturers in the development of their protective pads.

## INTRODUCTION

An intensive use of the bicycle can lead to nontraumatic injuries as reported by Dettori and Norvell.^1^ In the last few years scientific literature focused on the overuse injuries affecting the genitourinary tract because of their effect on the reduction of cyclists quality of life: mechanical causes of these injuries and countermeasures to reduce the problem are the main topics of researchers. Dettori et al^2^ examined the relationship between bicycle characteristics and the occurrence of erectile dysfunction in 463 cyclists who completed a cycling event of at least 320 km. Results showed a cumulative incidence of erectile dysfunction after the ride of 4.2% and of 1.8% 1 week and 1 month after the event. Leibovitch and Mor^3^ reported in a review that the most common bicycling associated urogenital problems are nerve entrapment syndromes presenting as genitalia numbness, which is reported in 50% to 91% of the cyclists, followed by erectile dysfunction reported in 13% to 24%. Schrader et al^4^ investigated the nocturnal penile tumescence and rigidity in 17 patrol officers riding an average of 5.4 h/day in comparison with 5 nonbiking men. Results showed that the percentage of sleep sessions that recorded an erectile event was significantly lower in the cyclists (27.1%) than it was in noncyclists (42.8%) suggesting that prolonged bicycle riding may have negative effects on nocturnal erectile function.

The compression and the stretch of the pudendal nerve near critical points like the ischiatic tuberosities and the pubic arc during pedaling seems to be the cause of both genital numbness and erectile dysfunction.^1,3,5–7^ The mechanical pressure leads to transient hypoxemia of the nerve and/or to a primary neuropathic process.^3,5^ The duration of these compressions seems to be more relevant than the amount of the pressure itself.^3,5^ Another possible pathophysiological mechanism of erectile dysfunction in cyclists is due to the compression of the perineal artery with a decrease of the blood flow to the penis and a consequent imbalance between the transforming growth factor beta 1 (TGF-β1) and prostaglandin E (PGE) in favor of TGF-b1 which induces collagen and connective tissue synthesis in the corpus cavernosum.^5^

Through examining this problem from a biomechanical point of view Gemery et al^8^ proposed a model of the complex saddle/perineum by means of tomography scans of 1 adult male pelvis and 3 bicycle seats. The pelvis/seats model developed by these authors showed that during cycling the most compressed point of the internal pudendal artery was immediately below the pubic symphysis.

The mechanical compression of perineum tissue during cycling was also investigated by means of magnetic resonance imaging of the perineal cavernous spaces, where the penile neurovascular tissues are located.^9^ This study confirmed the most inferior aspect of the pubic symphysis to be the most compressed part. Furthermore, corpus spongiosus diameter was 148% greater than the loaded condition while right and left corpora cavernosa diameters were, respectively, 232% and 252% with respect to the loaded condition. The evolution of bicycle saddle geometries has aimed to reduce the compressive load on the corresponding soft tissues. To evaluate the reduction in such loads pressure sensor mats resulted to be the more indicated systems and thus they have been employed in previous investigations.^10–13^ From a physiological point of view the capacity of different saddle shapes to reduce perineal pressure was indirectly quantified measuring the penile oxygen pressure.^7,14^ For this aim the most indicated instrument is the oxymeter. To provide evidence-based information in developing saddle design, Potter et al^15^ investigated the influence of gender, power, hand position, and ischial tuberosities width on saddle pressure during seated stationary cycling. Only 1 previous study^16^ has investigated the level of perceived comfort using different saddle shapes. The authors found that a partial nose saddle design may result in more comfort than a standard or complete no nose 1.

Surprisingly, even if within the cycling community it is well recognized the importance of padded shorts, scientific research on this topic is lacking. To the best of our knowledge, only 1 paper^17^ proposed the use of padded shorts as a preventive measure against skin chafing. On this point, an additional advice comes from the Royal Military College of Australia which stated that pants with chamois pads are the most comfortable and allow sweat absorption.^18^ Despite the paucity in the literature with regard to this topic, the beneficial effect of other padded cyclist garments such as gloves on reducing the pressure on the ulnar nerve was previously demonstrated.^19^

Therefore, the aim of this study was to analyze the effect of cyclist padded shorts in reducing pressure on the buttocks, with the ultimate goal of helping the manufacturers designing the pads. To accomplish this task, we evaluated 3 different types of pads introducing an objective evaluation of their level of comfort using biomechanical measurements, which was compared with the subjective ranking expressed by the cyclists.

## METHODS

### Participants

Nine male club road cyclists were enrolled in the study (age 26 ± 6 year; weight 66.5 ± 4.6 kg; height 175.0 ± 3.2 cm) following the Ansley and Cangley's cyclist categorization.^20^ Inclusion criteria involved absence of past and ongoing perineal numbness and of injuries to the back and lower limbs. All participants had a minimum cycling mileage of 10,000 km/year. These criteria were chosen to ensure that their body was perfectly familiarized to the posture imposed by their own bicycle. All participants read and signed an informed consent with the description of the testing procedures approved by the ethical committee of the Department of Biomedical Sciences, University of Padova.

### Instruments and Experimental Protocols

Each cyclist performed the test with his own bicycle fixed to a Real Power cycling roller (Elite, Fontaniva, PD, Italy). The system was connected to a notebook to collect speed and power output data at a sampling rate of 1 Hz. Saddle pressure measurement was obtained with the Pliance X-32 system (Novel, Munich, Germany) and its bicycle pressure mat (model S2019) composed of 234 square capacitive sensors (sensor dimension: 1.875 × 1.875 cm). The mat was calibrated to a maximum value of 400 kPa and the sampling rate was set at 60 Hz. It was placed with its median line coincident with the saddle median line and with its anterior margin aligned with the top of the saddle nose. A 6 camera infrared stereophotogrammetric system operating at 60 Hz (BTS Bioengineering, Italy, Garbagnate MIlanese, MI) was used to collect kinematic data of 22 passive reflective markers bilaterally placed in specific anatomical landmarks of the cyclist: acromion, elbow, wrist, great trochanter, posterior superior iliac spine, anterior superior iliac spine, lateral knee, fibula head, lateral malleolus, heel, and metatarsal head of the fifth toe. The anterior wheel of the bike was placed on a force plate (Bertec, Columbus, OH, USA) to collect force data during the sessions of pedaling.

The standardized warm up consisted of 5 min stretching exercises for lower limbs and 5 min of self-pace cycling. Subsequently, each participant was asked to perform 3 bouts of 20 min cycling wearing 1 of the 3 different padded shorts in each bout, assigned in a random order. The possible “shorts artifact error” due to the positioning of the 4 markers of the pelvis and of the great trochanters was avoided by cutting the cloth and applying the markers directly on the skin.

All participants had to maintain a speed of 30 km/h with the same gear (53:18); the hands were placed over the brake levers, simulating a realistic aerobic training pace. In order to better understand the behavior of the padding relative to the time spent on the saddle, cyclists had to pedal in a seated position during the whole test. Rest between trials was set to 5 to 7 min, representing the time necessary to change the padded shorts. Kinematic and pressure data were recorded for each pad at the 5th, 15th and 20th min period; a manual trigger was used for data synchronization. At the end of the test, each participant was asked to rate the most comfortable pad, as in the study conducted by Bressel and Larson.^16^

**FIGURE 1:**
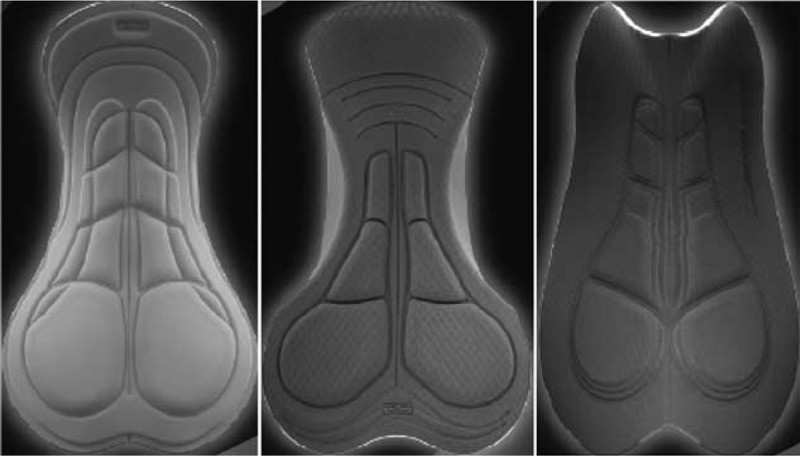
Pads stuck on the shorts worn by participants. From left to right: Blaze or BAS, Tour or INT, and Multi-D Anatomic or END. BAS = basic; INT = intermediate; END = endurance.

The 3 pads tested (Figure 1) in the study were: Blaze (345 × 210 mm, thickness of 5–10 mm with material density of 60 kg/m^3^) (basic, BAS), Tour (370 × 205 mm, thickness of 3–110 mm with material density of 80 kg/m^3^) (intermediate, INT), and Multi-D Anatomic (340 × 240 mm, thickness of 3–110 mm with material density of 80 and 120 kg/m^3^, respectively, front and back) (endurance, END). All the pads were produced by CyTech s.r.l Company (San Vendemiano, TV, Italy). BAS is a basic pad of the manufacturer, designed for short distances, INT is an intermediate model, and END is specifically developed for long distances and presents an anatomic curved shape. The participants’ bicycles were all equipped with a traditional narrow and flat competition saddle, without any kind of central hole. The choice of the same model was made for a better standardization of the research protocol even though no significant difference in penile oxygenation was observed when cycling on a saddle with or without central hole.^5^

### Data Analysis

Kinematic and pressure data were analyzed over a window of 29 normalized pedaling strokes for each of the 3 data recording periods (min 5, 15, and 20). One stroke was defined by 2 consecutive top dead centers through the identification of the vertical coordinate of the marker placed on the right metatarsal head of the 5th toe. This relatively high number of pedaling strokes had been chosen to obtain a consistent and repeatable amount of data allowing a better comparison of the 3 padded shorts.

#### Kinematics and Kinetics

**FIGURE 2:**
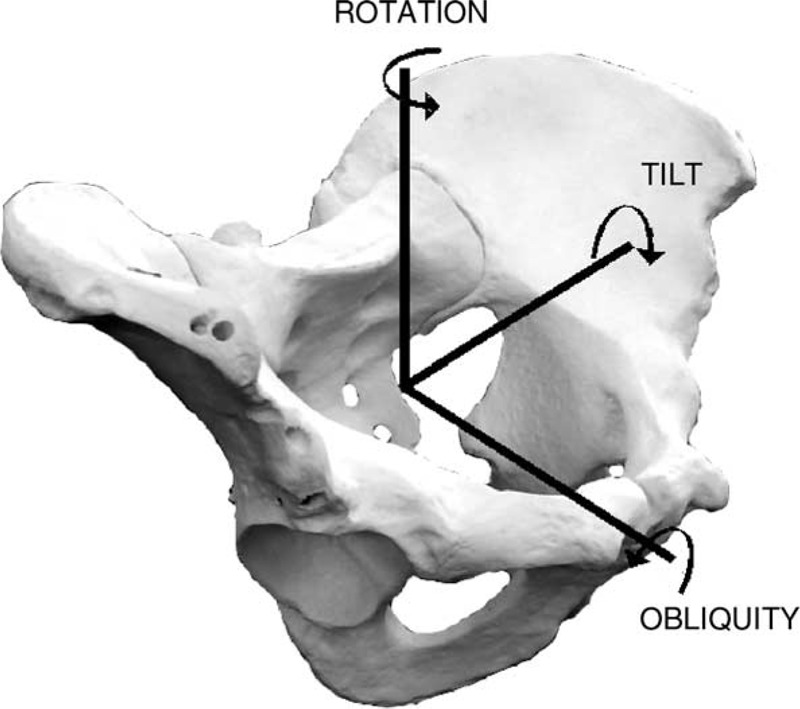
Pelvis reference system.

Kinematics was assessed through a customized tool in SMART Analyzer (BTS Bioengineering, Italy). Mean tilt values together with obliquity and rotation excursions were calculated and averaged among the 29 pedaling strokes for each padded shorts and for each data recording period. Positive values of pelvic tilt were referred to anteversion while negative values were referred to retroversion (Figure 2).

The relative angle between the plane of the pelvis and the trunk was also calculated and averaged among the 29 pedaling strokes for each padded shorts in order to provide information on the overall spine inclination.

The vertical component of the ground reaction force (vGRF) was measured as an objective comfort index, in order to evaluate possible changes in weight distribution of the cyclists in relationship with the different models of cycling shorts. We hypothesized that when the cyclist perceived a low level of comfort on the saddle, 1 of the countermeasures could be to reduce the discomfort by unloading the saddle, thus shifting forward his center of mass. Therefore, the load over the handlebars, and consequently on the anterior wheel, should increase. Furthermore, the maximum and the mean vGRF, over the normalized pedaling strokes were calculated and expressed as a percentage of the participants’ body weight.

#### Pressure Distribution on the Saddle

**FIGURE 3:**
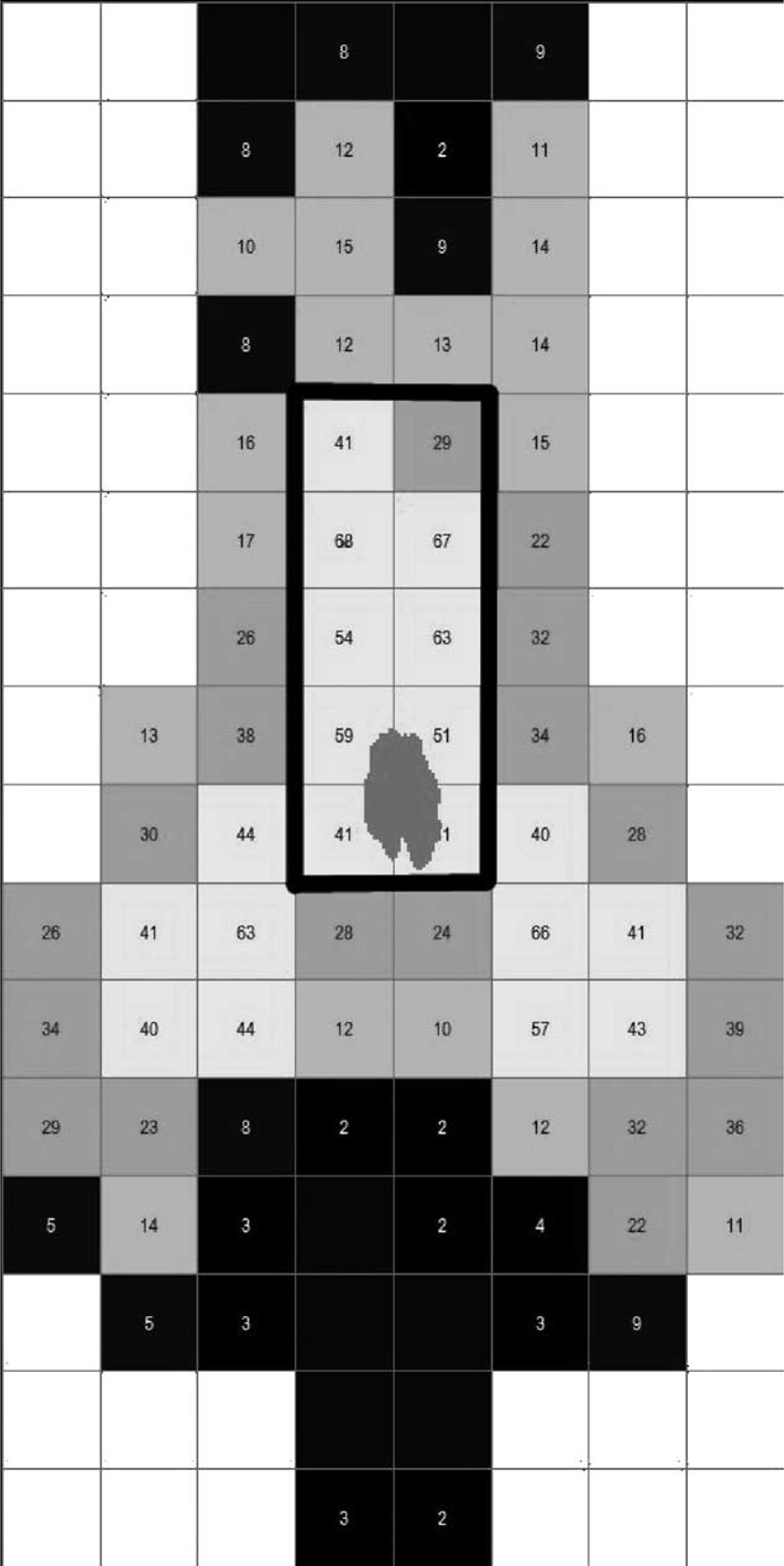
Saddle surface after the application of the software mask: the perineal area is represented by the cells included in the black rectangular.

Considering that the area of the saddle pressure mat was wider than the saddle area itself, a mask of sensors reproducing the overall contact area between the buttocks of the cyclists and the saddles was created with a customized software (Novel, Germany) (Figure 3). An average value among the same pedaling strokes considered for the kinematic analysis was calculated for the following variables: vertical force, mean pressure, and peak pressure. Vertical force was computed as the integral of the pressure applied to the saddle area frame by frame and normalized to the body weight of the cyclists; mean pressure was defined as the average of the pressure values among all the sensors of the mask and peak pressure indicated the highest value of pressure.

#### Center of Pressure (COP)

In order to obtain an accurate information of the load on the perineum, the trajectory of the COP was calculated from the pressure measurement system. A rectangular area determined from the first 2 cells including the COP and consisted of other 4 couples of cells toward the nose of the saddle was defined as the cyclists perineum (Figure 3). The area of each cell was 3.516 cm^2^ and the total perineum area was 35.16 cm^2^. Considering the 29 pedaling strokes, the absolute peak pressure of every cell was calculated and then the mean of those 10 values was used as the perineal mean peak pressure.

**FIGURE 4:**
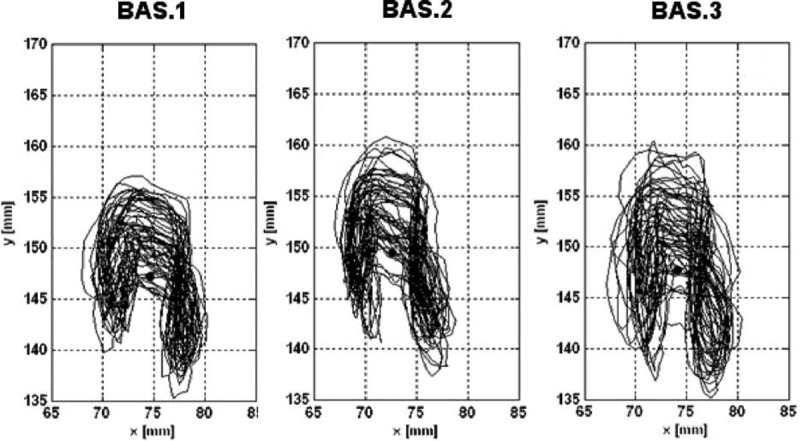
Example of center of pressure (COP) path for each data recording period. Example referred to 1 representative subject wearing pad BAS. BAS = basic.

The movements of the cyclist while pedaling seated over the saddle were hypothesized to be an objective comfort index for the evaluation of the level of comfort during pedaling. We propose that the increase in saddle movements would result in a higher level of discomfort, leading to longer length of the COP trajectory (Figure 4). Therefore, the length of the COP during the 29 pedaling strokes for each padded shorts and for each data recording period was calculated to investigate the interaction among padding and time on the level of comfort.

### Statistical Analysis

After data have been checked with the Kolmogorov–Smirnov normality test, a 2-way repeated measure ANOVA (type of pad × test duration) was used for statistical analysis of dependent variables. When F value showed interactions between the factors or effect of time, Bonferroni post hoc pair-wise comparison was calculated. Significant level for differences was set to *P* < 0.05. Data analysis was performed using the software package GraphPadPrism 4.00 (GraphPad Software, San Diego, CA).

## RESULTS

The overall mean velocity of the trials was 30 ± 0.4 km/h and the overall mean power was 140.1 ± 4.2 W. The results of the 3 data recording periods for each padded shorts were defined through the use of a number as a subscript. For example considering INT: INT.1 (data referred to min 5), INT.2 (data referred to min 15), and INT.3 (data referred to min 20).

### Kinematics and Pressure Distribution on the Saddle

**FIGURE 5:**
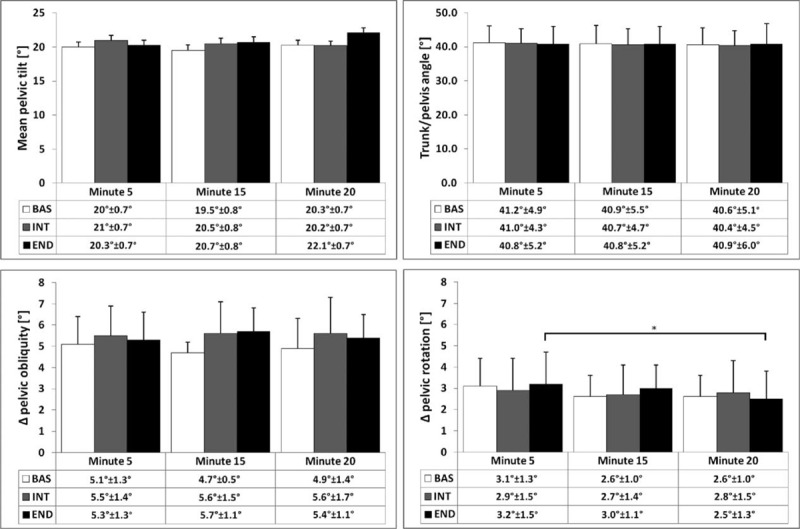
Pelvis kinematic results at minute 5, 15, and 20. For obliquity and rotation the differences among the maximum and minimum values were considered. Tilt represents the mean value of the 29 pedaling strokes. Data are presented as mean ± standard deviation (^∗^*P* < 0.05).

The analysis of pelvis kinematics showed a common behavior among cyclists; the widest angular excursion was the obliquity followed by the rotation. Also pelvic tilt angle and pelvis/trunk angle presented similar values with only small, nonsignificant, differences reported (Figure 5). Statistical analysis showed no interaction effect or main effect of time except for the pelvic rotation where a significant time effect was found (*P* = 0.0061). Post hoc tests showed a significant difference (*P* < 0.05) only between END.1 and END.3 with a reduction of pelvic rotation after 20 min of pedaling. In Table 1, mean values among the 29 pedaling strokes of peak pressure, mean pressure, and vertical force are reported for the 3 different periods of pedaling. No interaction effect between type of pad and time was found, but a main effect of time was detected (*P* < 0.0001). Vertical force decreased over time for BAS (BAS.1 vs BAS.2, *P* < 0.05; BAS.2 vs BAS.3, *P* < 0.05; BAS.1 vs BAS.3, *P* < 0.001) and END (END.1 vs END.3, *P* < 0.01) as well as mean pressure mean, for BAS (BAS.1 vs BAS.2, *P* < 0.05; BAS.2 vs BAS.3, *P* < 0.05; BAS.1 vs BAS.3, *P* < 0.001) and END (END.1 vs END.3, *P* < 0.01). The perineal mean peak pressure, referred to the cells representing the perineal area is presented in Table 2. Statistical analysis showed no interaction effect between type of pad and time; however, a significant time effect (*P* = 0.0124) was reported only for BAS (BAS.1 vs BAS.3, *P* < 0.05) after post hoc tests.

**TABLE 1:**
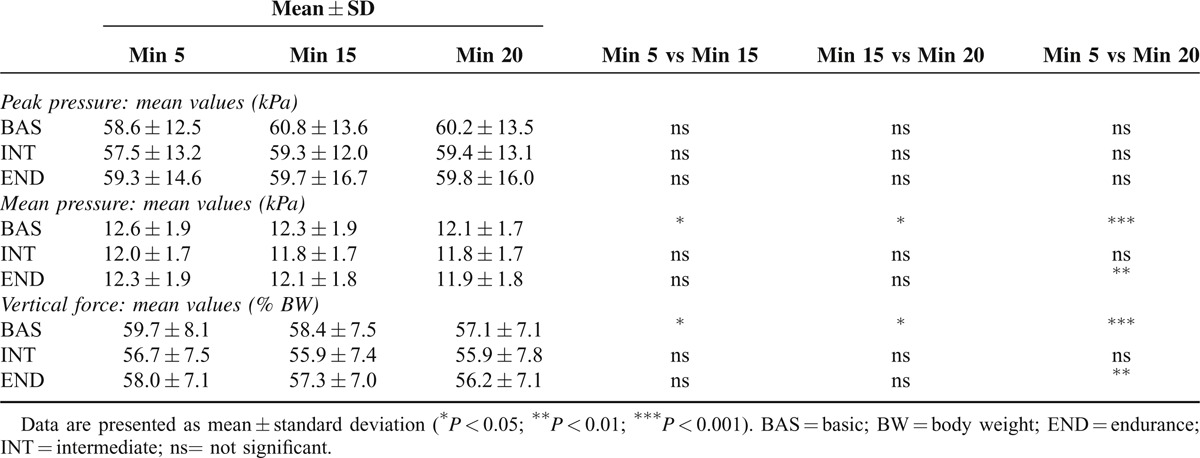
Mean Values Among the 29 Pedaling Strokes of Peak Pressure, Mean Pressure, and Vertical Force Considering the Saddle Surface and the 3 Periods of Pedaling

**TABLE 2:**
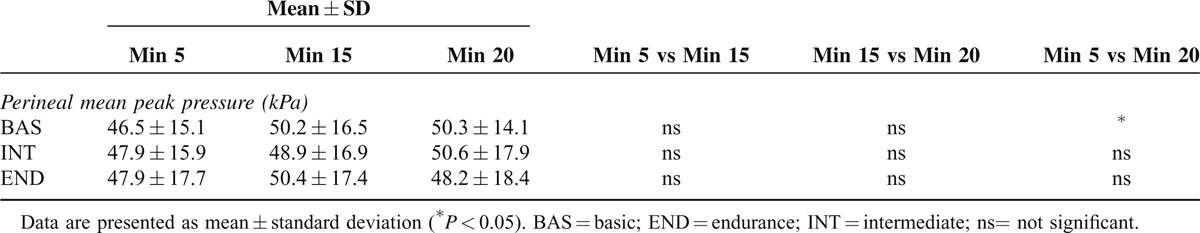
Comparison of the Perineal Mean Peak Pressure Determined Considering the COP Trajectory

### Comfort Evaluation

**FIGURE 6:**
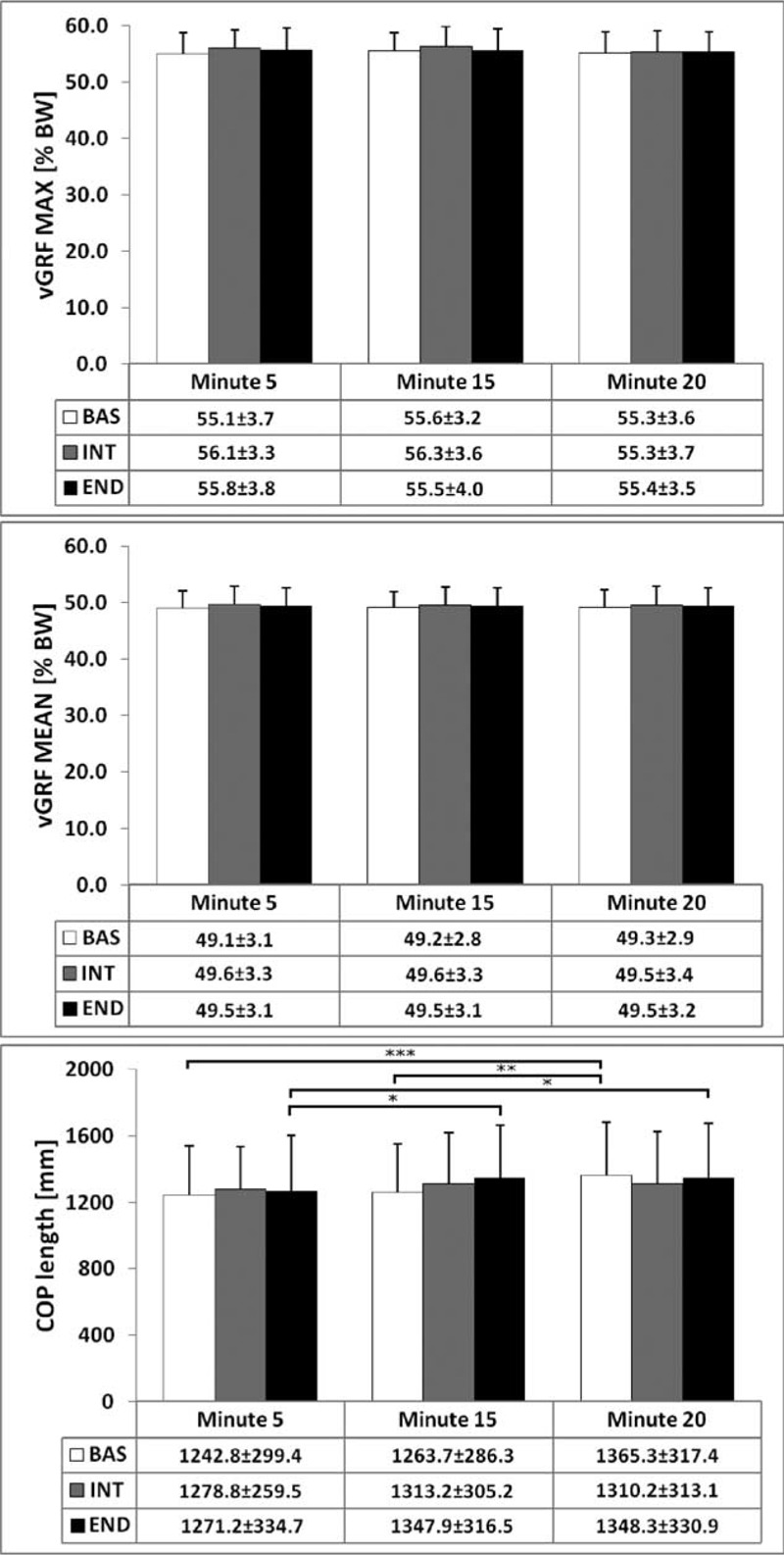
Comfort evaluation parameters: length of the center of pressure (COP), mean, and peak vGRF. Data are present as mean ± standard deviation (^∗^*P* < 0.05). vGRF = vertical component of the ground reaction force.

In regard to the length of the COP trajectory, both an interaction effect between type of pad and time and a main effect of time were recorded (*P* = 0.0451 and *P* < 0.0001, respectively). Post hoc tests showed a significant increase of this parameter for BAS (BAS.2 vs BAS.3, *P* < 0.01; BAS.1 vs BAS.3, *P* < 0.001) and END (END.1 vs END.2, *P* < 0.05; END.1 vs END.3, *P* < 0.05). vGRF data showed no statistically significant differences across test conditions (Figure 6). With regard to the subjective evaluation of the 3 padded shorts, 4 subjects preferred BAS, 4 subjects INT, and 1 subject END.

## DISCUSSION

A novice road cyclist needs a considerable amount of time to find the most convenient posture on the bike. This is because the trunk is flexed forward up to become almost parallel to the ground when hands are placed on the inferior part of the handlebars.^21,22^ Prolonged time spent in this posture could lead to muscle soreness, especially at the lumbar and cervical regions. A correct posture on the bike is fundamental in reducing these problems but it has to be accompanied by specific muscle training and long distance riding.^21^ For these reasons we selected club road cyclists with a consistent riding posture thus guaranteeing that possible modifications in the variables investigated were due to the effect of padded shorts and not concealed by fatigue caused by an unusual posture. Considering that all long distance road cyclists wear padded shorts, the present study, aimed to assess whether different pad design and material could have detectable effects on pressure distribution and level of comfort.

Saddle surface pressure values in the present study were smaller in magnitude compared with previous studies.^11,12^ This may be attributed to the weight of the participants recruited, which was less than the weight of the participants of the previous 2 studies.^11,12^ Moreover, in the experiments described by Lowe et al,^12^ the participants were asked to adjust the position of the ergometer handlebars to replicate the posture they adopted on their service bike, with less trunk flexion with respect to the road cyclists. In fact there is extensive evidence supporting the positioning of the trunk almost parallel to the road to maintain the most aerodynamic posture.^22^ Consequently, participants reported an increased saddle load and greater mean saddle pressure than shown by Bressel and Cronin^11^ because the posture on city bikes involved a reduced anterior trunk flexion. Pressure distribution data suggest an influence of the trunk forward position on the mechanical load of the perineal area and on the area below the pubic arc. However, this forward position did not increase the load on the posterior zone in correspondence of the ischiatic tuberosities. END pad offered the highest perineal protection, with an increase of perineal mean peak pressure after 20 min of only 0.6%, followed by INT (5.6%) and by BAS (8.2%). This loss of performance after 20 min of pedaling can be attributed to the compaction of pads’ material. We believe that the correct understanding of this phenomenon is fundamental for the health of long distance cyclists who spend several hours on their bike. Therefore, future studies should consider longer outdoor cycling sessions to better balance testing procedures with the amount of data collected.

The analysis of pelvis kinematics is in agreement with previous investigations,^16,23^ which found that the highest angular excursions occurred on the frontal and transversal plane (Figure 5). Nevertheless, the design and the material of the tested pads did not affect pelvis kinematics which is more influenced by the shape of the saddle.^16^ The only statistically significant difference was the pelvis rotation between END.1 and END.3. Considering that END is the only pad with 2 different densities (80 kg/m^3^ in the anterior part and 120 kg/m^3^ in the posterior one), we can speculate that its structure is responsible for this behavior. Nevertheless, it has to be mentioned that the magnitude of such an excursion is limited compared to the effect of the saddle shape.^16^ Results related to the angle between pelvis and trunk showed no statistically significant differences between the pads confirming the more pronounced effect of the saddle shape.^16^

The hypothesis whereby a higher level of discomfort corresponds to a larger weight on the anterior wheel was not confirmed by the measured vGRF. We speculate that the participants would sustain the perineal pain and the discomfort until they need to change posture by lifting the buttocks up from the saddle or moving backward/forward along it, rather than increasing the load on the anterior wheel. However, we cannot rule out the possibility that vGRF could have been mitigated by any upper body force acting on the handlebars.

In addition, the objective comfort index linked to the length of the COP trajectory did not find correspondence with the subjective comfort evaluations of cyclists. However, this could be explained by analyzing the characteristics of the 3 pads, which were designed for longer distances, but tested in rather short experimental trials. Furthermore, the general manufacturer recommendation was to utilize BAS for up to 2 h of pedaling, INT up to 3 h and END up to 6 h. This may be the reason why the cyclists selected BAS and INT as being the most comfortable. Moreover, we can speculate that 20 min of pedaling, even with the buttocks always on the saddle, was enough to test BAS and INT but not enough to completely stress the pad material of END, which was specifically designed for longer distances. Nevertheless, considering the increase of the COP length of the 3 padded shorts at the end of the trial (9.85% for BAS, 2.46% for INT, and 6.06% for END), we can speculate that BAS material was already starting to lose its elastic properties, due to its low foam density (60 kg/m^3^). This hypothesis could be supported also by the peak pressure data. Results of this study pointed out that the assessment of objective comfort index for cyclists’ padded shorts is a very complex task that cannot be linked to single parameters. Analogously, also the subjective evaluation should cover more specific aspects of the perceived comfort. We suggest that a matrix combining both these aspects can improve the characterization of the padded shorts with regard to comfort and protection. In regard to this, the unique question posed to the cyclists asking which was the best padded shorts tested has to be acknowledged as a limitation of the present study. Thus, future ergonomic investigations on subjective evaluation will have to explore its several aspects with visual analog scales and then integrate the results with biomechanical index measurements, similarly to what is being carried out on other bicycle components such as wheels.^24^

In conclusion, the present study indicates that the multifaceted topic of discomfort and genitalia numbness should consider not only the saddle geometry and the body posture, as extensively reported in scientific literature, but also the properties of the pads. Although our results support this statement, showing how the pad designed for END resulted to be the most efficient considering perineal peak pressure, further investigations are needed to explore the possible combined effects of different pads with different saddle designs and to develop objective comfort indexes that correlate to the cyclists’ subjective evaluations.
